# Supplementary material for: A network dynamical simulation model for the study of antibiotic resistance in microbial communities
Source: Gut Microbes. 2026 Sep 19;18(1):2734703. doi: 10.1080/19490976.2026.2734703 (PMC13596919; doi:10.1080/19490976.2026.2734703)
Supplement: Supplementary file 1 [file KGMI_A_2734703_SM9053.zip › supplementary_file_1/Figures/real_delta_n_components.pdf]

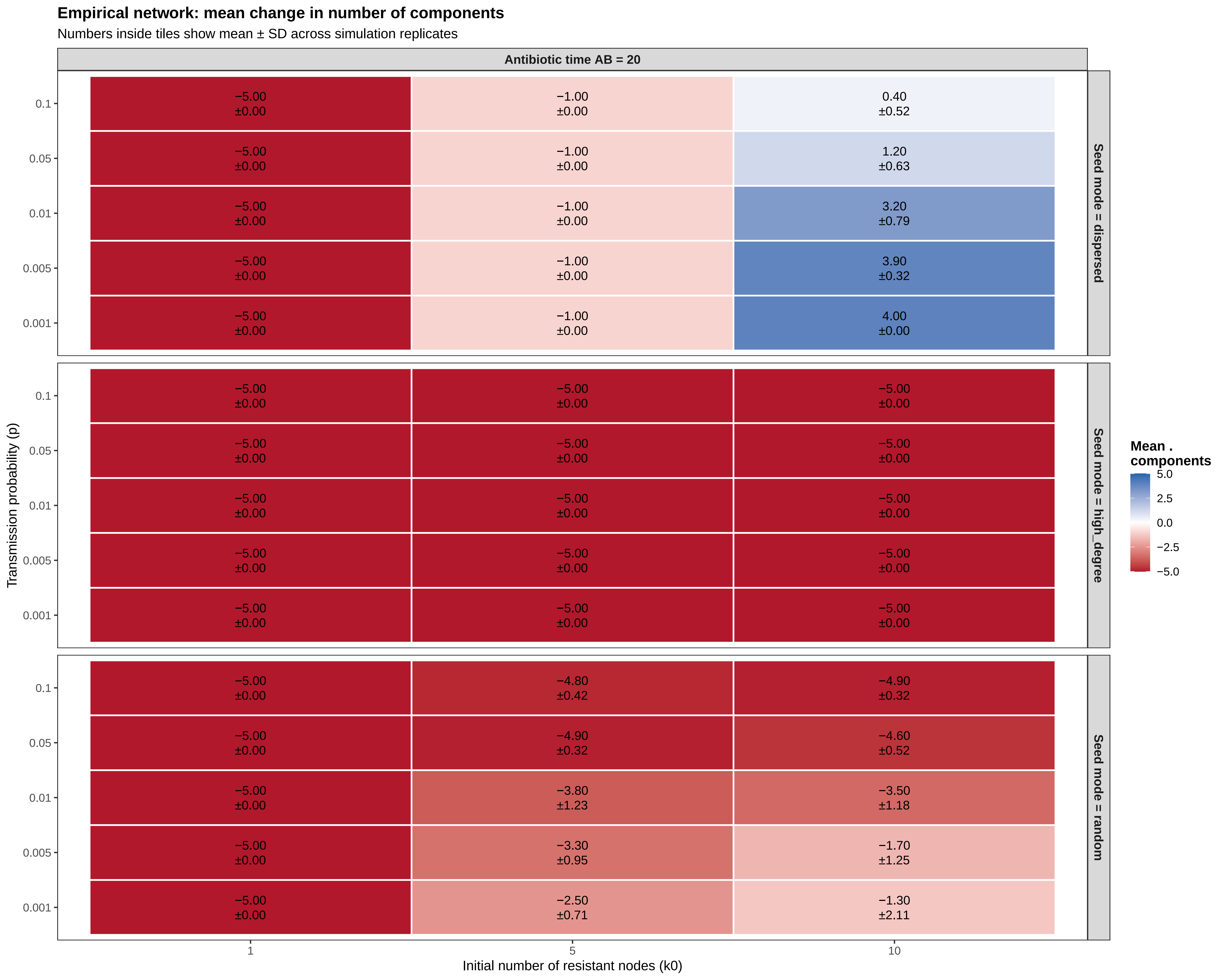
Empirical network: mean change in number of components
Numbers inside tiles show mean ± SD across simulation replicates
Antibiotic time AB = 20
Seed mode = dispersed
Seed mode = high_degree
Seed mode = random
Transmission probability (p)
0.1
0.05
0.01
0.005
0.001
Initial number of resistant nodes (k0)
1
5
10
Mean .
components
5.0
2.5
0.0
−2.5
−5.0
−5.00 ±0.00
−1.00 ±0.00
0.40 ±0.52
1.20 ±0.63
3.20 ±0.79
3.90 ±0.32
4.00 ±0.00
−4.80 ±0.42
−4.90 ±0.32
−4.90 ±0.32
−4.60 ±0.52
−3.80 ±1.23
−3.50 ±1.18
−3.30 ±0.95
−1.70 ±1.25
−2.50 ±0.71
−1.30 ±2.11
